# *In Vitro* Tolerance of Drug-Naive Staphylococcus aureus Strain FDA209P to Vancomycin

**DOI:** 10.1128/AAC.01154-16

**Published:** 2017-01-24

**Authors:** Madhuri Singh, Miki Matsuo, Takashi Sasaki, Yuh Morimoto, Tomomi Hishinuma, Keiichi Hiramatsu

**Affiliations:** Department of Microbiology, Faculty of Medicine, Juntendo University, Tokyo, Japan

**Keywords:** MSSA, *ileS*, killing rate, resistance, tolerance, vancomycin

## Abstract

The mechanisms underlying bacterial tolerance to antibiotics are unclear. A possible adaptation strategy was explored by exposure of drug-naive methicillin-susceptible Staphylococcus aureus strain FDA209P to vancomycin *in vitro*. Strains surviving vancomycin treatment (vancomycin survivor strains), which appeared after 96 h of exposure, were slow-growing derivatives of the parent strain. Although the vancomycin MICs for the survivor strains were within the susceptible range, the cytokilling effects of vancomycin at 20-fold the MIC were significantly lower for the survivor strains than for the parent strain. Whole-genome sequencing demonstrated that *ileS*, encoding isoleucyl-tRNA synthetase (IleRS), was mutated in two of the three vancomycin survivor strains. The IleRS Y723H mutation is located close to the isoleucyl-tRNA contact site and potentially affects the affinity of IleRS binding to isoleucyl-tRNA, thereby inhibiting protein synthesis and leading to vancomycin tolerance. Introduction of the mutation encoding IleRS Y723H into FDA209P by allelic replacement successfully transferred the vancomycin tolerance phenotype. We have identified mutation of *ileS* to be one of the bona fide genetic events leading to the acquisition of vancomycin tolerance in S. aureus, potentially acting via inhibition of the function of IleRS.

## INTRODUCTION

Staphylococcus aureus is one of the most important antibiotic-resistant pathogens that threaten global health in the 21st century (1). Penicillin-resistant S. aureus (PRSA) evolved soon after the introduction of penicillin G in 1943. The subsequent development of the penicillinase-resistant β-lactam antibiotic methicillin was followed by the evolution of methicillin-resistant S. aureus (MRSA) in 1961 in the United Kingdom. Since that time, MRSA outbreaks have been reported worldwide (2). MRSA isolates are often reported to be resistant to other classes of antibiotics, including vancomycin, leading to the emergence of vancomycin-intermediate S. aureus (VISA) (3–5).

Apart from resistance, a bacterial subpopulation can also survive under antibiotic pressure through another adaptive strategy, which is known as tolerance (6–8). Antibiotic tolerance has gained attention because of the ever increasing need to combat multidrug resistance as the severity of the antibiotic resistance crisis becomes more acute. Unlike resistance, alterations of antibiotic target sites and MICs are not typical hallmarks of tolerance (9, 10). Instead, slow growth and reduced autolysis have been reported to be common phenotypes of tolerant strains (7, 9, 10). Antibiotic-induced tolerance is reversible under drug-free conditions, when it is not accompanied by genetic mutation (10, 11). Although the specific molecular mechanisms of tolerance have not been determined for each class of antibiotic, results have revealed the presence of redundant molecular pathways that can lead to slow growth and the induction of tolerance (6). However, tolerance to cell wall-active antibiotics and other environmental stresses has been largely associated with the induction of the stringent response regulator guanosine tetra- and pentaphosphate [(p)ppGpp] (12). Whole-genome sequencing using next-generation sequencing (NGS) techniques is now being applied to obtain an understanding of tolerance mechanisms. By this method, daptomycin tolerance in S. aureus has been correlated with the occurrence of a point mutation in the *pitA* gene (encoding the inorganic phosphate transporter), which leads to the accumulation of inorganic phosphate and upregulation of the expression of the *dlt* operon, rather than with the induction of a stringent response (13, 14).

Because of the lack of a standard method of comparison, the identification of tolerant clinical bacterial isolates has been challenging (7). Nevertheless, tolerance may be an underlying cause of persistent infections and the recalcitrance of the bacteria to both host immunity and antibiotics.

Because vancomycin is an anti-MRSA agent, the genetic determinants responsible for the sequential conversion of MRSA to hetero-VISA (hVISA) and then to VISA under the selective pressure of vancomycin are well established both *in vitro* and *in vivo* (5, 15–23). However, we aimed to study the occurrence of vancomycin tolerance in a methicillin-susceptible S. aureus (MSSA) strain, to determine the early steps of the development of resistance. Understanding these early steps can potentially help to prevent the emergence of overt resistance, especially that associated with a gradual increase in the MICs of mainstay antibiotics, such as vancomycin (24–26).

In this study, strains surviving vancomycin treatment (referred to here as vancomycin survivor [VSV] strains) were selected from cultures of MSSA FDA209P exposed to an inhibitory concentration of vancomycin and were analyzed for physiological and genetic changes. We have previously published the complete genome sequence of FDA209P (27). Notably, this strain has no history of exposure to man-made antibiotics, as it was isolated in 1948, before their widespread clinical introduction (28). The use of this strain enabled exploration of the first steps in the adaptation of S. aureus to vancomycin, prior to the development of the capacity for multidrug resistance.

## RESULTS

### Establishment of vancomycin survivor mutants from FDA209P.

FDA209P cells (10^7^ CFU) were exposed to vancomycin at the MIC, leading to the appearance of three very small colonies after 96 h of incubation. Presumably, they resumed growth only after substantial consumption of vancomycin from the BHI agar plate (29). Unlike the growth of resistant mutants, the growth of these late-appearing survivors remained suppressed in the presence of an inhibitory concentration of vancomycin. Therefore, we speculated that these colonies were tolerant mutants and designated them vancomycin survivor strains VSV1 to VSV3 (Table 1). Next, the phenotypes and genotypes of these VSV strains were compared with those of reference strain FDA209P to understand the molecular mechanism of vancomycin tolerance.

**TABLE 1:** Description of bacterial strains and plasmids used in this study

Strain or plasmid	Description	Source or reference
Strains		
S. aureus strains		
FDA209P	Parental strain	28
VSV1, VSV2, VSV3	VSV strains with slow growth derived from FDA209P exposed to vancomycin at 1.5 mg/liter	This study
FDA209P-ileS*	FDA209P into which the *ileS* Y723H mutation was introduced by the allelic replacement method	This study
E. coli DH5α	Used for molecular cloning	TaKaRa Bio, Shiga, Japan
Plasmids		
pKOR1	E. coli-S. aureus shuttle vector used for allelic exchange	46
pKOR-ileS*	pKOR1 plasmid carrying a portion of the *ileS* gene with the *ileS* Y723H mutation (ileS*)	This study

### Vancomycin-tolerant phenotype of vancomycin survivor strains.

**FIG 1:**
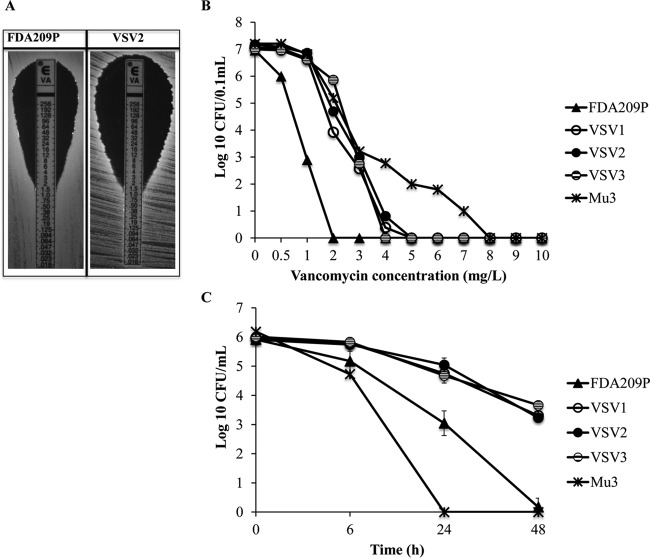
Vancomycin-tolerant phenotype of the vancomycin survivor strains (strains VSV1 to VSV3) derived from FDA209P upon single exposure to 1.5 mg/liter vancomycin. (A) Evaluation of vancomycin MIC by Etest of strain VSV2 and parental strain FDA209P. The MIC was 1.5 mg/liter for both strains. (B) Population analysis on vancomycin exposure of three VSV strains, parent strain FDA209P, and the hetero-vancomycin-intermediate S. aureus strain Mu3. (C) Cytokilling curves of S. aureus strains on exposure to 30 mg/liter vancomycin (20× MIC). The numbers of CFU per milliliter were determined at the indicated times. Vancomycin tolerance was indicated by an increase in the MDK_99.9_. Data are presented as the means ± SDs from three independent experiments.

The vancomycin susceptibility of the FDA209P-derived survivor strains was determined by Etest (Table 2). MIC values were <2 mg/liter (Fig. 1A), which is below the susceptibility breakpoint of 4 mg/liter for vancomycin, indicating a slight reduction in susceptibility, but these cells were not as resistant as VISA cells.

**TABLE 2:** Phenotypic and genotypic properties of the VSV strains derived from FDA209P

Strain	Growth rate		Vancomycin susceptibility			Nonsynonymous SNP(s)
	DT^*d*^ (min)	Lag time (h)	MIC^*a*^ (mg/liter)	MBC^*b*^ (mg/liter)	MDK_99.9_^*c*^ (h)	
FDA209P	30.8	3	1.5	30	24	
VSV1	61.0	5	1.5	>30	>48	*divIVA* D54Y
VSV2	59.4	5	1.5	>30	>48	*ribA* M40I, *ileS* Y723H
VSV3	66.6	5	1	>30	>48	*ileS* A196V

a MICs were evaluated after 48 h of incubation.

b MBC, minimum bactericidal concentration required to kill ≥3 log_10_ CFU (≥99.9% killing) within 24 h.

c MDK_99.9_, minimum duration of 99.9% killing.

d DT, doubling time.

To further evaluate the change in vancomycin susceptibility of the survivor strains, we compared the population analysis patterns (the number of CFU at different concentrations of vancomycin) of the survivor strains with those of control strains, including FDA209P and hVISA Mu3 (Fig. 1B). Mu3 demonstrated heteroresistant colonies even in the presence of higher concentrations of vancomycin up to 8 mg/liter, whereas no resistant subpopulation was observed in any of the survivor strains growing in the presence of ≥4 mg/liter vancomycin. Nevertheless, all survivor strains demonstrated a modest increase in colony counts with vancomycin concentrations of 2 to 3 mg/liter, resulting in a shift of the population curves to the right compared with the curve for FDA209P.

By definition, tolerant mutants have a lower rate of antibiotic-induced cytokilling than the parental strain (10). The levels of cytokilling of the survivor mutants were compared with those of FDA209P and hVISA Mu3 to determine the tolerance to vancomycin. The cytokilling activity of vancomycin (20× MIC) against the survivor strains was substantially reduced and slower than that against FDA209P and Mu3 (Fig. 1C and Table 2). With FDA209P, 30 mg/liter vancomycin resulted in a reduction in the log_10_ number of CFU of >3 (bactericidal effect) within 24 h of incubation (24), so the minimum duration of killing of 99.9% of the cell population (MDK_99.9_) was 24 h. However, the viable counts of VSV1, VSV2, and VSV3 were 3.30 ± 0.13, 3.23 ± 0.16, and 3.66 ± 0.04 log_10_ CFU/ml, respectively, on treatment with vancomycin for 48 h, indicating that the MDK_99.9_ values were >48 h. Notably, the cytokilling curve for Mu3 was steeper than the curves for strains VSV1 to VSV3 and FDA209P, indicating that although Mu3 is a hVISA strain, it consists of a population that is homogeneously sensitive to vancomycin at 30 mg/liter and that is 100% killed within 24 h, whereas the populations of strains VSV1 to VSV3 were heterogeneously sensitive to vancomycin at 30 mg/liter and had subpopulations of 0.1 to 0.3% that still survived at 48 h.

The difference in the log_10_ number of CFU per milliliter between variants VSV1 to VSV3 and parental strain FDA209P was found to be significant (*P* < 0.05) at 24 h and 48 h of incubation.

### Additional features of vancomycin-tolerant survivor strains.

**FIG 2:**
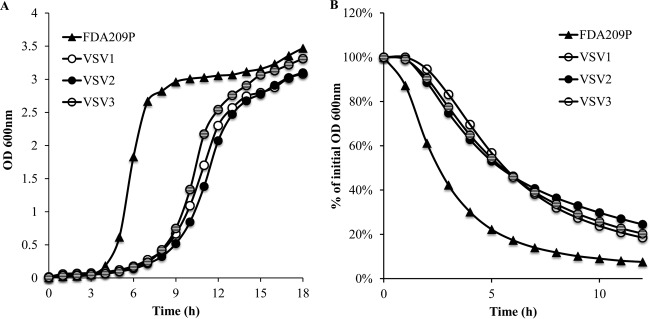
Additional features of VSV strains. (A) Growth rate. Stationary-phase cultures were grown and diluted to ∼10^5^ CFU/ml in fresh BHI broth, and the growth rate was measured by recording the OD_600_ every 2 min over 24 h. Strains VSV1 to VSV3 were slow growing, showing an increased doubling time and a prolonged lag phase relative to those of FDA209P. (B) Autolytic activity. Strains VSV1 to VSV3 underwent autolysis at a substantially lower rate than parent strain FDA209P. Data are representative of those from three independent experiments with reproducible observations.

Other physiological changes associated with vancomycin tolerance were analyzed by measuring the growth rates and autolytic rates for the survivor strains relative to those for strain FDA209P. Slow growth was observed for all the survivor strains, with the doubling time being increased (up to 70 min) and the lag phase being prolonged (up to 5 h) compared with those for FDA209, which grew with a doubling time of 30 min and which had a lag phase of 2.5 to 3 h (Table 2 and Fig. 2A).

The Triton X-100-induced autolysis of strains VSV1 to VSV3 and FDA209P was also compared, and a marked decrease in the autolytic rates in all the VSV strains was demonstrated (Fig. 2B). It is possible that cell wall thickening is the cause of the reduced autolysis activity (30). Taken together, the slow growth and reduced autolysis of strains VSV1 to VSV3 were further indications of tolerance, as both of these phenotypes have previously been associated with antibiotic-induced tolerance (7, 9, 10).

### Identification of SNPs in the vancomycin survivor strains.

**FIG 3:**
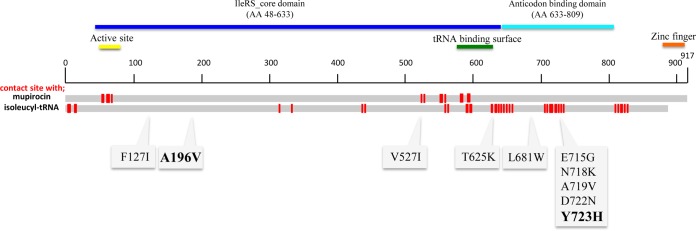
Mapping of *ileS* mutations and contact sites of tRNA^Ile^ and mupirocin on the domains of isoleucyl-tRNA synthetase (IleRS). The amino acid (AA) location and the alteration of all the unique nonsynonymous single-nucleotide polymorphisms identified by *ileS* gene sequencing of vancomycin-tolerant mutants are shown in the boxes. Point mutations of *ileS* that were confirmed by whole-genome sequencing are indicated in bold. The contact sites of tRNA^ile^ and mupirocin on IleRS are indicated.

To determine the genetic basis of tolerance, the whole-genome sequences of strains VSV1 to VSV3 were compared with the whole-genome sequence of FDA209P, and nonsynonymous single-nucleotide polymorphism (SNPs) were identified in each case (Table 2). Two of the three VSV mutants had a single nonsynonymous SNP in the *ileS* (SAFDA_1063) gene (encoding IleRS). One SNP resulted in a change at residue 196 of IleRS from Ala to Val, and the other resulted in a change at residue 723 from Tyr to His (Fig. 3). Residues 196 and 723 are located in very close proximity to the active site and isoleucyl-tRNA contact sites of IleRS, respectively (see Table S2 in the supplemental material). According to structural and evolutionary predictions, residue 723 is buried within the three-dimensional structure of IleRS (http://homcos.pdbj.org/?LANG=ja) (Table S3). Moreover, amino acid residue 723 varies among IleRS homologues, indicating a tendency for this residue to change under different environmental conditions. On the contrary, residue 196A seems to be conserved among IleRS homologues. Nonsynonymous SNPs were also identified in two other genes: *ribA* (SAFDA_1653), encoding an enzyme involved in riboflavin biosynthesis, and *divIVA* (SAFDA_1062), encoding cell division initiation protein DivIVA. The *ribA* SNP was identified in VSV2, and the *divIVA* SNP was identified in VSV1. The reproducibility of *ileS* mutant selection under vancomycin pressure was also confirmed by Sanger sequencing using 12 colonies from three plates; 11 of the colonies had point mutations concentrated from residues 500 to 723 of the IleRS protein. The specific locations of these mutations are shown in Fig. 3.

### Mupirocin, a direct IleRS inhibitor, induces vancomycin tolerance in FDA209PA.

**FIG 4:**
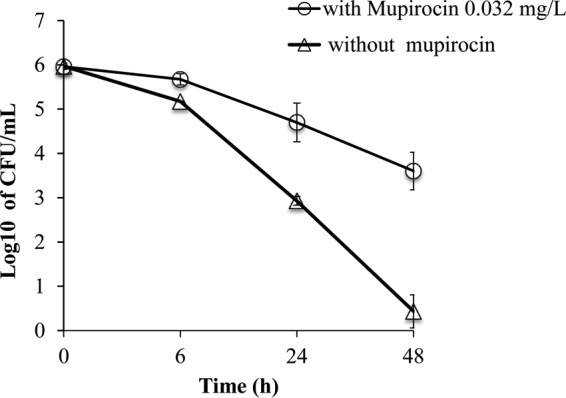
Mupirocin-induced vancomycin tolerance in wild-type strain FDA209P. Cytokilling curves of FDA209P were produced after exposure to vancomycin at 20× MIC in BHI broth supplemented with 0.032 mg/liter mupirocin or without mupirocin. The values are presented as the means ± SDs from two independent experiments.

One of the two *ileS* mutations detected in the VSV strains was located at residue 723, which is in close proximity to the isoleucyl-tRNA contact site, leading us to speculate that a reduction in binding affinity between isoleucyl-tRNA and IleRS Y723H (compared with that for wild-type IleRS) might have induced vancomycin tolerance. To test this hypothesis, the effect of mupirocin, a direct inhibitor of IleRS (31), on vancomycin cytokilling efficacy against the FDA209P strain was tested. Cytokilling by vancomycin took longer in the presence of a sub-MIC of mupirocin, and FDA209P showed tolerance against vancomycin at 20× MIC, with the count of the survivors being ∼10^3^ CFU/ml at 48 h (Fig. 4). This result suggests that *ileS* mutations in vancomycin survivor strains act like mupirocin by inhibiting IleRS function, resulting in protein synthesis inhibition and thereby causing vancomycin tolerance.

### Replacement of wild-type IleRS with IleRS Y723H replicated vancomycin tolerance in FDA209P.

**FIG 5:**
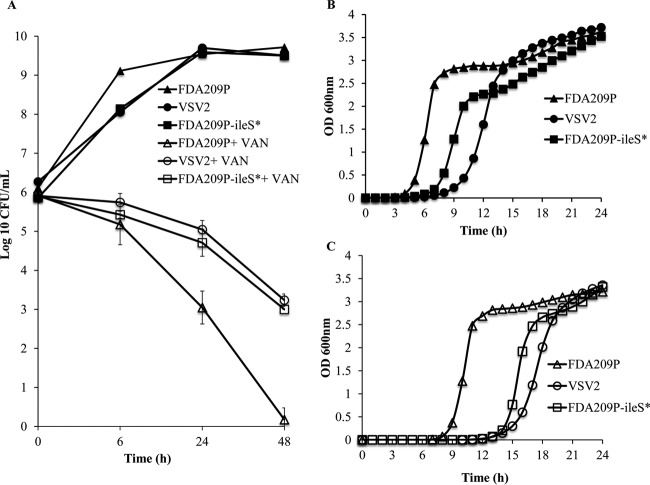
Association of the *ileS* Y723H mutation with vancomycin (VAN) tolerance and low growth rate. (A) Cytokilling curves of ileS* mutants, including vancomycin survivor VSV2 and FDA209P-ileS*, indicate tolerance to vancomycin. Cells were treated with 30 mg/liter vancomycin for >48 h, and the numbers of CFU per milliliter were measured at the indicated times. The MDK_99.9_ was calculated. Values are presented as the means ± SDs from three independent experiments. (B) Growth rates of ileS* mutants and FDA209P. Drug-free stationary-phase cultures were grown and diluted to ∼10^5^ CFU/ml in fresh BHI broth without vancomycin, and the growth rate was measured by recording the OD_600_ every 2 min over 24 h. The lag phase was defined as the period during which the OD_600_ remained static, and the doubling time was extracted from the fit to the exponential phase. (C) Resuscitation of ileS* mutants and FDA209P after vancomycin exposure. Drug-free stationary-phase cultures (10^6^ CFU/ml) were treated with 30 mg/liter vancomycin for 6 h, followed by washing and inoculation (10^5^ CFU/ml) in drug-free BHI broth to measure the growth rate over 24 h. After vancomycin exposure, the ileS* mutants resumed growth at a much lower rate than wild-type strain FDA209P. The growth rates shown are representative of those from three independent experiments with similar results.

The experimental results of mupirocin-induced vancomycin tolerance in FDA209P indirectly suggested a close association between inhibition of IleRS and the occurrence of the tolerance phenotype of VSV2 resulting exclusively from the *ileS* mutation and not the *ribA* mutation. To directly demonstrate the association between the *ileS* Y723H mutation and vancomycin tolerance, this mutation was generated in wild-type FDA209P by pKOR1-mediated allelic replacement (Table 1). The constructed FDA209P strain into which the *ileS* Y723H mutation was introduced by the allelic replacement method, FDA209P-ileS*, was evaluated for vancomycin sensitivity and growth patterns (Fig. 5A to C). Similar to VSV2, the cytokilling rate of FDA209P-ileS* was significantly lower than that of FDA209P in the presence of 30 mg/liter vancomycin at all time points, with ∼0.1% of the initial population (10^3^ CFU/ml) being alive at 48 h (Fig. 5A). Therefore, the MDK_99.9_ of FDA209P-ileS* was 48 h, which is 24 h longer than that of FDA209P. The growth rate of FDA209P-ileS* before and after exposure to vancomycin was also investigated. The growth rate of FDA209P-ileS* was lower than that of FDA209P even without vancomycin exposure, with the doubling time being 45.9 ± 0.8 min and the lag period being 4 h (Fig. 5B). The growth rate was measured after exposure to 30 mg/liter vancomycin for 6 h. Vancomycin exposure increased the lag phase in all strains (Fig. 5C) compared with that before vancomycin exposure (Fig. 5B). The increase in the lag phase with vancomycin exposure was greater in FDA209P-ileS* and VSV2 than in FDA209P. Taken together, these results indicate that the ileS* mutation replicated the vancomycin tolerance phenotypes in FDA209P, demonstrating that *ileS* mutations are responsible for inducing vancomycin tolerance.

### Cross-tolerance of *ileS* Y723H mutants to other antibiotics.

**FIG 6:**
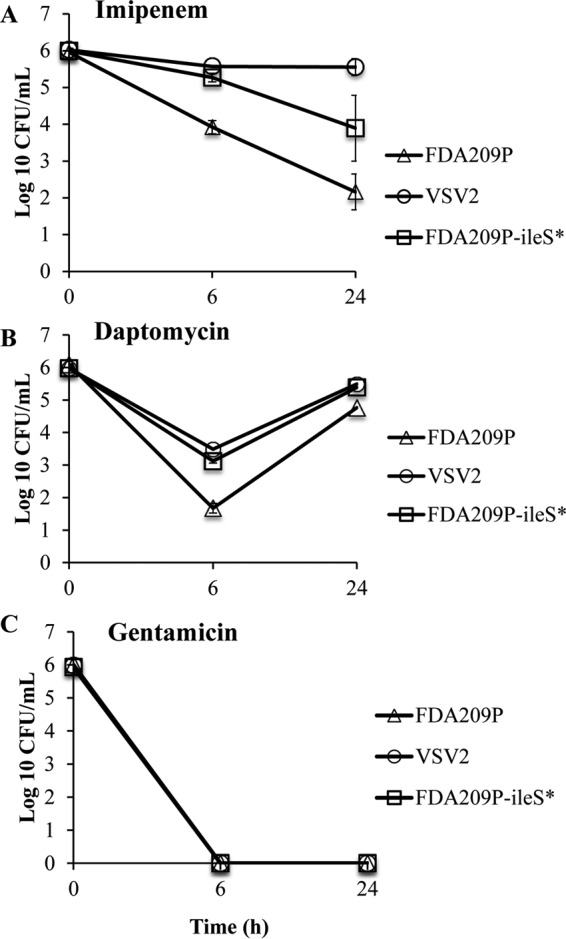
Cross-tolerance of *ileS* Y723H mutants to other antibiotics. Cytokilling curves of *ileS* Y723H mutants (VSV2 and FDA209P-ileS*) and parental strain FDA209P were obtained after exposure to 20× MIC of imipenem (A), daptomycin (B), and gentamicin (C). The values are presented as the means ± SDs from two independent experiments.

The sensitivity of the *ileS* Y723H mutants to four other antibiotics belonging to different classes, imipenem (a β-lactam), daptomycin (a lipopeptide), gentamicin (an aminoglycoside), and mupirocin (an IleRS inhibitor), was tested by both MIC and time-kill assays. The MIC of each of these antibiotics for *ileS* Y723H mutants was less than the susceptibility breakpoint (data not shown). The cytokilling curves of the study strains for imipenem, daptomycin, and gentamicin at 20× MICs are shown in Fig. 6A to C, respectively. Both VSV2 and FDA209P-ileS* showed tolerance to imipenem, as indicated by increased colony counts compared with those for FDA209P (Fig. 6A). These *ileS* mutants were also tolerant to daptomycin (Fig. 6B). However, the *ileS* mutation did not reduce the sensitivity to gentamicin, which showed a bactericidal effect within 6 h of treatment (Fig. 6C). The results suggest that the *ileS* mutation contributes to increased tolerance to antibiotics that target the cell envelope but not to gentamicin, a protein synthesis inhibitor. These results suggest that use of the combination of vancomycin with gentamicin might be useful to avoid the emergence of antibiotic tolerance.

## DISCUSSION

Previous studies on antibiotic tolerance have suggested that a low growth rate and reduced autolysis are physiological characteristics of mutants with tolerance to cell wall-acting antibiotics (7, 10, 32). The slow-growing phenotype of these mutants has been explained by the occurrence of the stringent response in association with *relA*/*spoT* homologous genes, which encode proteins that synthesize a global stress regulator, (p)ppGpp (12, 33, 34). The accumulation of (p)ppGpp ultimately brings the cell into a slow-growing state that shields it from the activity of antibiotics by inactivating their targets (12, 35, 36). In contrast, daptomycin tolerance is not related to the (p)ppGpp-mediated stringent response or to a slow-growth phenotype. Instead, daptomycin tolerance correlates with upregulation of the *dlt* operon, which is induced by the accumulation of inorganic phosphate, suggesting that antibiotic-specific molecular pathways also lead to tolerance (13, 14). In this study, we sought to identify genetic changes in surviving bacteria following exposure of an antibiotic-naive S. aureus strain to vancomycin at the MIC.

Although the MICs for the vancomycin survivor strains (strains VSV1 to VSV3) were not increased relative to those for FDA209P (Fig. 1A), the more sensitive population analysis showed a clear shift of the curves to the right, indicating the presence of subpopulations with increased resistance. Notably, however, the survivor strains showed increased tolerance to vancomycin-induced killing compared with that of the FDA209P and Mu3 (hVISA) strains (Fig. 1C). Even though it had a low vancomycin MIC, the MSSA strain FDA209P exhibited a high tolerance which exceeded that of the hVISA strain Mu3, suggesting that tolerance and resistance are quite different phenomena (10). In the future, in addition to resistance based on MIC values, tolerance based on cytokilling rates may be applied to tests for the clinical diagnosis of bacterial infections (26). In addition, our results suggest that not only MRSA strains but also MSSA strains could be recalcitrant to anti-MRSA agents, such as vancomycin and daptomycin, probably because of the property of tolerance. Moreover, the vancomycin survivor strains showed cotolerance to other antibiotics, including imipenem and daptomycin (Fig. 6). Therefore, the use of a combination of two antibiotics with different mechanisms might be helpful to avoid the emergence of vancomycin tolerance.

From our results, along with those presented in earlier reports, it seems probable that slow growth is a feature of tolerance, irrespective of the stress types and bacterial genera (9, 33). A similar combination of slow growth and resistance to killing may be observed in a naturally occurring biofilm of bacteria, whose foundation is based on a dormant, drug-tolerant subpopulation which is recalcitrant to multidrug therapy and responsible for persistent infections (6, 32, 35).

Whole-genome comparisons of tolerant mutants identified *ileS*, encoding IleRS, to be one of the key genes involved in vancomycin tolerance. Moreover, IleRS belongs to the aminoacyl-tRNA synthetases and is an essential enzyme in protein synthesis (37). Therefore, it is very likely that direct or indirect impairment of one or more amino acyl-tRNA synthetases would initiate (p)ppGpp-mediated stringent responses by creating amino acid starvation. Previously, the inactivation of glutamyl-tRNA synthetase in a *hipA* (high persister) mutant derived from Escherichia coli was reported to induce β-lactam tolerance via a (p)ppGpp-mediated mechanism (34).

The *ribA* and *divIVA* mutations were also detected in VSV strains with and without an *ileS* mutation, respectively. *divIVA* encodes the DivIVA protein, which has an essential role in the initiation of septum formation for cell division (38). Independently (or perhaps synergistically, in the case of *ileS* and *ribA*), these mutations are likely to have enabled cells to cope with compromised cellular fitness under vancomycin pressure. Furthermore, introduction of the IleRS Y723H variant in the reference FDA209P strain demonstrated the association between ileS* and tolerance phenotypes, including a reduced killing rate and a prolonged lag phase compared with those for the wild type (Fig. 5A to C).

This study represents the first identification of mutations in the *ileS*, *divIVA*, and *ribA* genes as part of the early step of MSSA adaptation to vancomycin. These mutations caused a considerable proportion (0.1 to 0.3%) of cells of three vancomycin-tolerant strains to be alive even after 48 h exposure to vancomycin at 20× MIC, which may lead to the recurrence of infection and the eventual failure of vancomycin therapy.

(p)ppGpp-mediated stringent responses are triggered by cell wall-active antibiotics, including vancomycin and β-lactams (33, 39, 40). An inhibitory concentration of vancomycin can induce (p)ppGpp accumulation through stimulation of *relA* in Enterococcus faecalis (33). In addition, *relA*-mediated stringent responses are triggered under the amino acid starvation created by mupirocin, which competitively inhibits the IleRS enzyme (33, 41). In our experiments with FDA209P on the vancomycin exposure plate, it was likely that all colonies that appeared after 96 h, including those with normal and small morphologies, were able to survive the vancomycin pressure under the double induction of the stringent response created by vancomycin itself and the *ileS* mutation. Of all of the survivors, only genetic mutants further retained the vancomycin tolerance phenotype after drug-free subculturing. Survivors without relevant mutations presumably lost the tolerance phenotype upon cessation of vancomycin exposure. Therefore, vancomycin might have induced reversible tolerance in wild-type survivors by modifying gene expression, causing a temporary stringent response. In contrast, induction of the stringent response in mutated survivor strains was stably inherited.

As the IleRS Y723H mutation in the VSV2 strain is located in close proximity to the isoleucyl-tRNA contact site, this mutation might affect the stable binding of isoleucyl-tRNA to IleRS, causing a reduced rate of synthesis of isoleucine–isoleucyl-tRNA and leading to the accumulation of uncharged isoleucyl-tRNA (33). RelA senses uncharged isoleucyl-tRNA and induces the stringent response by synthesizing (p)ppGpp (12, 40). This stress mediator acts on RNA polymerase and changes the physiology of the cell, blocking protein synthesis, inhibiting cell division, and causing slow growth (6, 35). In this situation, cell wall synthesis is not required for survival and vancomycin becomes nonlethal (6, 12).

This study provides direct evidence for the involvement of the mutation of IleRS in the acquisition of a vancomycin tolerance phenotype, along with tolerance to other cell wall-acting antibiotics. Tolerance is closely linked to a low growth rate (especially prolongation of the lag phase) and is presumably associated with the continuous expression of the stringent response. Further studies using transcriptomic and proteomic analyses, along with direct measurement of the levels of (p)ppGpp in ileS* mutants, are needed to prove this hypothesis.

## MATERIALS AND METHODS

### Bacterial strains and growth conditions.

FDA209P (ATCC 6538P) was the parent strain used for the selection of vancomycin-tolerant S. aureus strains. The derived strains, along with the plasmids used in this study, are described in Table 1. S. aureus was grown in brain heart infusion (BHI) broth (Becton, Dickinson and Company, Sparks, MD, USA) with aeration at 37°C, unless indicated otherwise, whereas Escherichia coli was grown in Luria-Bertani medium (LB; Becton, Dickinson and Company). To select E. coli and S. aureus transformants, 100 mg/liter ampicillin or 10 mg/liter chloramphenicol, respectively, was added to the growth medium. Antibiotics were purchased from Sigma (St. Louis, MO, USA).

### Selection of vancomycin-tolerant strains.

Vancomycin-tolerant strains were selected after inoculation of 10^7^ CFU of a stationary-phase culture of FDA209P on BHI agar plates containing 1.5 mg/liter vancomycin (equivalent to the MIC for FDA209P), and the appearance of survivors was monitored up to 120 h. Three very small colonies that appeared after 96 h of incubation were selected for further analysis. These three colonies were streaked and grown on drug-free BHI agar plates. A single colony was then picked from each of the three cultures, followed by growth in BHI broth. The resulting cultures were established as representative vancomycin survivor strains (strains VSV1, VSV2, and VSV3) (Table 1). The isogenicity of the survivor strains to their parent strain was confirmed by multilocus sequence typing (MLST).

### Measurement of growth rate.

The growth rate parameters, including the lag phase and doubling time, were measured as described elsewhere (20). Briefly, a portion of an overnight culture was grown to an optical density (OD) at 600 nm (OD_600_) of 0.3, and then a 10-μl aliquot of this culture was inoculated into 10 ml fresh BHI broth (final concentration, 10^5^ CFU/ml). This culture was grown at 37°C with shaking at 25 rpm in an automatic photorecording incubator (model TN-2612; Advantec, Tokyo, Japan). The OD_600_ was recorded every 2 min over a period of 24 h. A growth curve was produced by plotting the OD against time. The lag phase was defined as the period showing no increase in the OD (static OD), whereas the doubling time of each strain was determined by fitting a growth curve to an exponential equation.

For postantibiotic lag-phase determination, stationary-phase drug-free cultures of the study strains were adjusted to 10^6^ CFU/ml in BHI broth containing 30 mg/liter vancomycin and incubated for 6 h at 37°C with shaking, and then 0.1-ml aliquots were centrifuged. The precipitated cells were washed with saline, resuspended in 0.1 ml BHI medium, and used to inoculate 10 ml BHI medium. The lag time and doubling time were measured as described above (14). The experiments were repeated at least three times on different days.

### Antibiotic susceptibility testing.

MICs were determined by the Etest gradient strip method (AB Biodisk, Solna, Sweden), following the manufacturer's instructions. BHI agar was used instead of Mueller-Hinton agar, as BHI agar is a proven medium for supporting VISA phenotype expression (20). The MIC was recorded after both 24 h and 48 h of incubation.

### Cytokilling assay.

The cytokilling assay was performed as described previously (42), with slight modifications. Briefly, overnight stationary-phase cultures of the test strains were adjusted to an OD_578_ of 0.3 (10^8^ CFU/ml). Next, 100 μl of this suspension was inoculated into 10 ml of prewarmed BHI broth (giving a final cell density of 10^6^ CFU/ml) either without vancomycin or with 30 mg/liter vancomycin (20× MIC) in glass tubes. These tubes were incubated with shaking at 37°C in an automated OD recorder. At 0 h, 6 h, 24 h, and 48 h, a 100-μl aliquot of the culture was harvested, diluted in saline solution, and spread on BHI agar plates. Viable colonies were counted after incubation of the plates at 37°C for 48 h to ensure the appearance of all colonies of the slow-growing strains. The log_10_ number of CFU was plotted against time for each treatment. The lowest vancomycin concentration that caused a reduction of the log_10_ number of CFU of ≥3 (≥99.9% killing) was defined as the minimum bactericidal concentration (MBC) (24). For parental control strain FDA209P, a time-kill assay was performed with a range of vancomycin concentrations (1×, 2×, 5×, 10×, and 20× MIC). The vancomycin MBC for the FDA209P parental control strain was 30 mg/liter (20× MIC), so the tolerance of the vancomycin survivor strains to vancomycin was tested at this concentration. All the cytokilling experiments were repeated at least three times.

### Population analysis.

Analysis of heteroresistant subpopulations was performed as described previously (3). Briefly, 100-μl aliquots of an overnight culture adjusted to an OD_578_ of 0.3, along with serial 10-fold dilutions, were plated on BHI agar containing from 0.5 mg/liter to 10 mg/liter vancomycin. The plates were incubated at 37°C for 24 to 72 h, and the number of colonies was counted. The population curve was drawn after plotting the log_10_ number of CFU versus the vancomycin concentrations.

### Autolysis assay.

Triton X-100-induced autolysis activity was measured as described previously (43). Briefly, cultures were grown to an OD_600_ of ∼2 with shaking at 37°C in an automatic OD recorder (model TN-2612). The cells were harvested at 4°C and added to prechilled 10 mM Tris-HCl (pH 7.5) containing 0.05% (vol/vol) Triton X-100, giving a final turbidity of ∼2 at 600 nm. A decrease in the OD_600_ resulting from autolysis was recorded during overnight incubation at 30°C, using an automatic biophotorecorder. The autolysis was reported as a percentage of the initial OD (at the zero time point), and final results are representative of those from three independent experiments.

### Whole-genome sequencing and determination of mutations.

The whole-genome sequences of the VSV1, VSV2, and VSV3 strains were compared with the whole-genome sequence of parental reference strain FDA209P using a next-generation sequencing (NGS) platform (Illumina MiSeq; Illumina Inc., San Diego, CA, USA). Briefly, a single colony from a streaked plate was grown to stationary phase in BHI broth, genomic DNA was isolated, and a DNA library was prepared using a Nextera XT DNA sample preparation kit (Illumina). Sequencing was performed using a MiSeq reagent kit (v2; 500 cycles) and a paired-end 2 × 250-bp cycle run on an Illumina MiSeq sequencing system.

In order to detect genetic alterations in the survivor strains, the 250-bp paired-end sequencing reads were mapped to the complete reference genome sequence of parental strain FDA209P (27) using the programs snpTree (v1.1) and CSI Phylogeny (v1.1) (44). The average percentage of the reference genome mapped was 99.94%, indicating that the number of reads in this analysis was sufficient.

The detected single-nucleotide polymorphisms (SNPs) were verified by resequencing of candidate genes by a Sanger sequencing method using a BigDye Terminator (v3.1) cycle sequencing kit on a 3500xL genetic analyzer (Applied Biosystems, Tokyo, Japan) with forward and reverse primers specific for each target locus. Structural and evolutionary information for proteins affected by nonsynonymous SNPs was searched for using the protein database HOMOCOS (http://homcos.pdbj.org/?LANG=ja).

### Recombinant DNA techniques.

Plasmid and genomic DNA was isolated using Miniamp and Miniprep kits (Qiagen, Valencia, CA, USA), respectively. Restriction enzymes were used as recommended by the manufacturer (TaKaRa Bio, Shiga, Japan). PCR amplification was performed using TaKaRa *Ex Taq* DNA polymerase and buffer (TaKaRa Bio).

The preparation of the recombinant plasmid pKOR1-ileS* and its transformation into chemically competent E. coli DH5α cells (TaKaRa) were performed as described previously (45). The recombinant plasmids (Table 1) were isolated from E. coli using midiprep kits (Qiagen), and the presence of intact insert DNA was verified by sequencing using the primers listed in Table S1 in the supplemental material, followed by electroporation into S. aureus by pulsating at 2,500 V using a Gene Pulser system (Bio-Rad, Hercules, CA, USA).

### Introduction of *ileS* mutation encoding isoleucyl-tRNA synthetase (IleRS) Y723H into FDA209P by pKOR1 allelic replacement.

The replacement of wild-type *ileS* (SAFDA_1063) in parental strain FDA209P was performed by the pKOR1 allelic replacement method, as described previously (46). Briefly, a 1.2-kb *ileS* DNA fragment (ileS*) from the VSV2 mutant (expressing IleRS Y723H) was amplified using primers attB1_ileS (1)_F and attB2_ileS (1)_R2 (Table S1). The PCR product (with an *attB* site at each end) was used for recombination with pKOR1, yielding the pKOR-ileS* plasmid. The sequencing integrity of the construct was confirmed by sequencing. This plasmid was then used in the allelic replacement procedure. The resultant ileS*-containing strain was designated FDA209P-ileS*.

### Statistical analysis.

The data from all the cytokilling experiments, which were repeated on three separate occasions, are presented as the mean ± standard deviation (SD). The significance of the differences in the level of cytokilling was determined by a paired Student's *t* test. A *P* value of <0.05 was considered significant.

## Supplementary Material

Supplemental material

## References

[1] Prestinaci F, Pezzotti P, Pantosti A 2015 Antimicrobial resistance: a global multifaceted phenomenon. Pathog Glob Health 109:309–318. doi:10.1179/2047773215Y.0000000030. 26343252 PMC4768623

[2] Chambers HF, Deleo FR 2009 Waves of resistance: *Staphylococcus aureus* in the antibiotic era. Nat Rev Microbiol 7:629–641. doi:10.1038/nrmicro2200. 19680247 PMC2871281

[3] Hiramatsu K, Aritaka N, Hanaki H, Kawasaki S, Hosoda Y, Hori S, Fukuchi Y, Kobayashi I 1997 Dissemination in Japanese hospitals of strains of *Staphylococcus aureus* heterogeneously resistant to vancomycin. Lancet 350:1670–1673. doi:10.1016/S0140-6736(97)07324-8. 9400512

[4] Mwangi MM, Wu SW, Zhou Y, Sieradzki K, de Lencastre H, Richardson P, Bruce D, Rubin E, Myers E, Siggia ED, Tomasz A 2007 Tracking the in vivo evolution of multidrug resistance in *Staphylococcus aureus* by whole-genome sequencing. Proc Natl Acad Sci U S A 104:9451–9456. doi:10.1073/pnas.0609839104. 17517606 PMC1890515

[5] Howden BP, McEvoy CR, Allen DL, Chua K, Gao W, Harrison PF, Bell J, Coombs G, Bennett-Wood V, Porter JL, Robins-Browne R, Davies JK, Seemann T, Stinear TP 2011 Evolution of multidrug resistance during *Staphylococcus aureus* infection involves mutation of the essential two component regulator WalKR. PLoS Pathog 7:e1002359. doi:10.1371/journal.ppat.1002359. PMC3213104 22102812

[6] Lewis K 2010 Persister cells. Annu Rev Microbiol 64:357–372. doi:10.1146/annurev.micro.112408.134306. 20528688

[7] Tuomanen E, Durack DT, Tomasz A 1986 Antibiotic tolerance among clinical isolates of bacteria. Antimicrob Agents Chemother 30:521–527. doi:10.1128/AAC.30.4.521. 3539006 PMC176473

[8] Dhar N, McKinney JD 2007 Microbial phenotypic heterogeneity and antibiotic tolerance. Curr Opin Microbiol 10:30–38. doi:10.1016/j.mib.2006.12.007. 17215163

[9] Fridman O, Goldberg A, Ronin I, Shoresh N, Balaban NQ 2014 Optimization of lag time underlies antibiotic tolerance in evolved bacterial populations. Nature 513:418–421. doi:10.1038/nature13469. 25043002

[10] Gefen O, Balaban NQ 2009 The importance of being persistent: heterogeneity of bacterial populations under antibiotic stress. FEMS Microbiol Rev 33:704–717. doi:10.1111/j.1574-6976.2008.00156.x. 19207742

[11] Haaber J, Friberg C, McCreary M, Lin R, Cohen SN, Ingmer H 2015 Reversible antibiotic tolerance induced in *Staphylococcus aureus* by concurrent drug exposure. mBio 6:e02268-14. doi:10.1128/mBio.02268-14. PMC4313918 25587013

[12] Maisonneuve E, Gerdes K 2014 Molecular mechanisms underlying bacterial persisters. Cell 157:539–548. doi:10.1016/j.cell.2014.02.050. 24766804

[13] Mechler L, Herbig A, Paprotka K, Fraunholz M, Nieselt K, Bertram R 2015 A novel point mutation promotes growth phase-dependent daptomycin tolerance in *Staphylococcus aureus*. Antimicrob Agents Chemother 59:5366–5376. doi:10.1128/AAC.00643-15. 26100694 PMC4538524

[14] Mechler L, Bonetti EJ, Reichert S, Flotenmeyer M, Schrenzel J, Bertram R, Francois P, Gotz F 2016 Daptomycin tolerance in the *Staphylococcus aureus pitA6* mutant is due to upregulation of the *dlt* operon. Antimicrob Agents Chemother 60:2684–2691. doi:10.1128/AAC.03022-15. 26883712 PMC4862447

[15] Matsuo M, Cui L, Kim J, Hiramatsu K 2013 Comprehensive identification of mutations responsible for heterogeneous vancomycin-intermediate *Staphylococcus aureus* (hVISA)-to-VISA conversion in laboratory-generated VISA strains derived from hVISA clinical strain Mu3. Antimicrob Agents Chemother 57:5843–5853. doi:10.1128/AAC.00425-13. 24018261 PMC3837870

[16] Matsuo M, Hishinuma T, Katayama Y, Hiramatsu K 2015 A mutation of RNA polymerase β′ subunit (RpoC) converts heterogeneously vancomycin-intermediate *Staphylococcus aureus* (hVISA) into “slow VISA.” Antimicrob Agents Chemother 59:4215–4225. doi:10.1128/AAC.00135-15. 25941225 PMC4468653

[17] Saito M, Katayama Y, Hishinuma T, Iwamoto A, Aiba Y, Kuwahara-Arai K, Cui L, Matsuo M, Aritaka N, Hiramatsu K 2014 “Slow VISA,” a novel phenotype of vancomycin resistance, found in vitro in heterogeneous vancomycin-intermediate *Staphylococcus aureus* strain Mu3. Antimicrob Agents Chemother 58:5024–5035. doi:10.1128/AAC.02470-13. 24841271 PMC4135821

[18] Katayama Y, Murakami-Kuroda H, Cui L, Hiramatsu K 2009 Selection of heterogeneous vancomycin-intermediate *Staphylococcus aureus* by imipenem. Antimicrob Agents Chemother 53:3190–3196. doi:10.1128/AAC.00834-08. 19451283 PMC2715608

[19] Cui L, Neoh HM, Shoji M, Hiramatsu K 2009 Contribution of vraSR and graSR point mutations to vancomycin resistance in vancomycin-intermediate *Staphylococcus aureus*. Antimicrob Agents Chemother 53:1231–1234. doi:10.1128/AAC.01173-08. 19124662 PMC2650561

[20] Matsuo M, Hishinuma T, Katayama Y, Cui L, Kapi M, Hiramatsu K 2011 Mutation of RNA polymerase beta subunit (*rpoB*) promotes hVISA-to-VISA phenotypic conversion of strain Mu3. Antimicrob Agents Chemother 55:4188–4195. doi:10.1128/AAC.00398-11. 21746940 PMC3165293

[21] Passalacqua KD, Satola SW, Crispell EK, Read TD 2012 A mutation in the PP2C phosphatase gene in a *Staphylococcus aureus* USA300 clinical isolate with reduced susceptibility to vancomycin and daptomycin. Antimicrob Agents Chemother 56:5212–5223. doi:10.1128/AAC.05770-11. 22850507 PMC3457403

[22] Gardete S, Kim C, Hartmann BM, Mwangi M, Roux CM, Dunman PM, Chambers HF, Tomasz A 2012 Genetic pathway in acquisition and loss of vancomycin resistance in a methicillin resistant *Staphylococcus aureus* (MRSA) strain of clonal type USA300. PLoS Pathog 8:e1002505. doi:10.1371/journal.ppat.1002505. PMC3271070 22319446

[23] Ishii K, Tabuchi F, Matsuo M, Tatsuno K, Sato T, Okazaki M, Hamamoto H, Matsumoto Y, Kaito C, Aoyagi T, Hiramatsu K, Kaku M, Moriya K, Sekimizu K 2015 Phenotypic and genomic comparisons of highly vancomycin-resistant *Staphylococcus aureus* strains developed from multiple clinical MRSA strains by in vitro mutagenesis. Sci Rep 5:17092. doi:10.1038/srep17092. 26603341 PMC4658547

[24] Sader HS, Becker HK, Moet GJ, Jones RN 2010 Antimicrobial activity of daptomycin tested against *Staphylococcus aureus* with vancomycin MIC of 2 microg/mL isolated in the United States and European hospitals (2006-2008). Diagn Microbiol Infect Dis 66:329–331. doi:10.1016/j.diagmicrobio.2009.09.017. 20159377

[25] Gould IM 2013 Treatment of bacteraemia: meticillin-resistant *Staphylococcus aureus* (MRSA) to vancomycin-resistant *S. aureus* (VRSA). Int J Antimicrob Agents 42(Suppl):S17–S21. doi:10.1016/j.ijantimicag.2013.04.006. 23664580

[26] Claeys KC, Lagnf AM, Hallesy JA, Compton MT, Gravelin AL, Davis SL, Rybak MJ 2016 Pneumonia caused by methicillin-resistant *Staphylococcus aureus*: does vancomycin heteroresistance matter? Antimicrob Agents Chemother 60:1708–1716. doi:10.1128/AAC.02388-15. 26729497 PMC4775950

[27] Singh M, Sasaki T, Matsuo M, Morimoto Y, Aiba Y, Hiramatsu K 2015 Complete genome sequence of the drug-naive classical *Staphylococcus aureus* strain FDA209P. Genome Announc 3(6):e01343-15. doi:10.1128/genomeA.01343-15. PMC4972786 26564052

[28] Shaw C, Stitt JM, Cowan ST 1951 Staphylococci and their classification. J Gen Microbiol 5:1010–1023. doi:10.1099/00221287-5-5-1010. 14908038

[29] Hiramatsu K, Katayama Y, Matsuo M, Aiba Y, Saito M, Hishinuma T, Iwamoto A 2014 Vancomycin-intermediate resistance in *Staphylococcus aureus*. J Glob Antimicrob Resist 2:213–224. doi:10.1016/j.jgar.2014.04.006. 27873679

[30] Cui L, Tominaga E, Neoh HM, Hiramatsu K 2006 Correlation between reduced daptomycin susceptibility and vancomycin resistance in vancomycin-intermediate *Staphylococcus aureus*. Antimicrob Agents Chemother 50:1079–1082. doi:10.1128/AAC.50.3.1079-1082.2006. 16495273 PMC1426436

[31] Silvian LF, Wang J, Steitz TA 1999 Insights into editing from an Ile-tRNA synthetase structure with tRNA^Ile^ and mupirocin. Science 285:1074–1077. doi:10.1126/science.285.5430.1074. 10446055

[32] Gilbert P, Collier PJ, Brown MR 1990 Influence of growth rate on susceptibility to antimicrobial agents: biofilms, cell cycle, dormancy, and stringent response. Antimicrob Agents Chemother 34:1865–1868. doi:10.1128/AAC.34.10.1865. 2291653 PMC171955

[33] Abranches J, Martinez AR, Kajfasz JK, Chavez V, Garsin DA, Lemos JA 2009 The molecular alarmone (p)ppGpp mediates stress responses, vancomycin tolerance, and virulence in *Enterococcus faecalis*. J Bacteriol 191:2248–2256. doi:10.1128/JB.01726-08. 19168608 PMC2655485

[34] Germain E, Roghanian M, Gerdes K, Maisonneuve E 2015 Stochastic induction of persister cells by HipA through (p)ppGpp-mediated activation of mRNA endonucleases. Proc Natl Acad Sci U S A 112:5171–5176. doi:10.1073/pnas.1423536112. 25848049 PMC4413331

[35] Shah D, Zhang Z, Khodursky A, Kaldalu N, Kurg K, Lewis K 2006 Persisters: a distinct physiological state of E coli. BMC Microbiol 6:53. doi:10.1186/1471-2180-6-53. 16768798 PMC1557402

[36] Chua SL, Yam JK, Hao P, Adav SS, Salido MM, Liu Y, Givskov M, Sze SK, Tolker-Nielsen T, Yang L 2016 Selective labelling and eradication of antibiotic-tolerant bacterial populations in *Pseudomonas aeruginosa* biofilms. Nat Commun 7:10750. doi:10.1038/ncomms10750. 26892159 PMC4762895

[37] Berg JM, Tymoczko JL, Stryer L 2002 Aminoacyl-transfer RNA synthetases read the genetic code. Section 29.2. *In* Biochemistry, 5th ed WH Freeman & Co, New York, NY.

[38] Edwards DH, Errington J 1997 The Bacillus subtilis DivIVA protein targets to the division septum and controls the site specificity of cell division. Mol Microbiol 24:905–915. doi:10.1046/j.1365-2958.1997.3811764.x. 9219999

[39] Mwangi MM, Kim C, Chung M, Tsai J, Vijayadamodar G, Benitez M, Jarvie TP, Du L, Tomasz A 2013 Whole-genome sequencing reveals a link between beta-lactam resistance and synthetases of the alarmone (p)ppGpp in *Staphylococcus aureus*. Microb Drug Resist 19:153–159. doi:10.1089/mdr.2013.0053. 23659600 PMC3662374

[40] Geiger T, Kastle B, Gratani FL, Goerke C, Wolz C 2014 Two small (p)ppGpp synthases in *Staphylococcus aureus* mediate tolerance against cell envelope stress conditions. J Bacteriol 196:894–902. doi:10.1128/JB.01201-13. 24336937 PMC3911181

[41] Traxler MF, Summers SM, Nguyen HT, Zacharia VM, Hightower GA, Smith JT, Conway T 2008 The global, ppGpp-mediated stringent response to amino acid starvation in *Escherichia coli*. Mol Microbiol 68:1128–1148. doi:10.1111/j.1365-2958.2008.06229.x. 18430135 PMC3719176

[42] Aiba Y, Katayama Y, Hishinuma T, Murakami-Kuroda H, Cui L, Hiramatsu K 2013 Mutation of RNA polymerase beta-subunit gene promotes heterogeneous-to-homogeneous conversion of beta-lactam resistance in methicillin-resistant *Staphylococcus aureus*. Antimicrob Agents Chemother 57:4861–4871. doi:10.1128/AAC.00720-13. 23877693 PMC3811421

[43] Gustafson JE, Berger-Bachi B, Strassle A, Wilkinson BJ 1992 Autolysis of methicillin-resistant and -susceptible *Staphylococcus aureus*. Antimicrob Agents Chemother 36:566–572. doi:10.1128/AAC.36.3.566. 1320363 PMC190558

[44] Kaas RS, Leekitcharoenphon P, Aarestrup FM, Lund O 2014 Solving the problem of comparing whole bacterial genomes across different sequencing platforms. PLoS One 9:e104984. doi:10.1371/journal.pone.0104984. PMC4128722 25110940

[45] Matsuo M, Kurokawa K, Lee BL, Sekimizu K 2010 Shuttle vectors derived from pN315 for study of essential genes in *Staphylococcus aureus*. Biol Pharm Bull 33:198–203. doi:10.1248/bpb.33.198. 20118540

[46] Bae T, Schneewind O 2006 Allelic replacement in *Staphylococcus aureus* with inducible counter-selection. Plasmid 55:58–63. doi:10.1016/j.plasmid.2005.05.005. 16051359

