# Supplementary material for: In Vitro Tolerance of Drug-Naive Staphylococcus aureus Strain FDA209P to Vancomycin
Source: Antimicrob Agents Chemother. 2017 Jan 24;61(2):e01154-16. doi: 10.1128/AAC.01154-16 (PMC5278750; doi:10.1128/AAC.01154-16)
Supplement: Supplemental material [file supp_61_2_e01154-16__index.html]

In Vitro Tolerance of Drug-Naive Staphylococcus aureus Strain FDA209P to Vancomycin — Supplemental material 

# *In Vitro* Tolerance of Drug-Naive Staphylococcus aureus Strain FDA209P to Vancomycin

## Supplemental material

- Supplemental file 1 -

  Tables S1 to S3

  PDF, 76K
