# Supplementary material for: In Vitro Tolerance of Drug-Naive Staphylococcus aureus Strain FDA209P to Vancomycin
Source: Antimicrob Agents Chemother. 2017 Jan 24;61(2):e01154-16. doi: 10.1128/AAC.01154-16 (PMC5278750; doi:10.1128/AAC.01154-16)
Supplement: Supplemental material [file AAC.01154-16_zac002175857s1.pdf]

TABLE S1 List of primers used and designed in this study

Name	Sequence (5' to 3')	Length/source
ribA_CP1	TGCACCTTCAAGTAATCTTCCA	22/ This study
ribA_CP2	TTGGTTTTTAGGAGGGGTAGC	21/ This study
ribA_SP1	TGGCATTGATATTGCAGAAAG	21/ This study
ileS_CP1	CACAGCGAATTAGGTAATGGTG	22/ This study
ileS_CP2	TGACCGGTAACTTGGTCATTT	21/ This study
divIV_CP1	TCCAAGGTTTTGGTAGAGCA	20/ This study
divIV_CP2	CACCATTACCTAATTCGCTGTG	22/ This study
attB1_ileS(1)_F	GGGGACAAGTTTGTACAAAAAAGCAGGCTCCAATATCGTGGTTGGTTCA	49/ This study
attB2_ileS(1)_R2	GGGGACCACTTTGTACAAGAAAGCTGGGTTGACCGGTAACTTGGTCATTT	50/ This study

Note: attB sites are underlined

TABLE S2 Description of contact sites of Isoleucyl-tRNA and Mupirocin on the domains of ileS-tRNA synthetase (IleRS)

Contacting molecule	Contact sites	source
Mupirocin	56P 57P 58Y 64H 66G 67H 70N 528W 531S 554E 555G 557D 558Q 562W 585H 586G 587F 588V 596M 597S 598K	Homocos
Isoleucyl-tRNA	3Y 4K 7L 9M 16M 17R 314H 315V 333D 436W 439T 440R 443N 556S 557D 560R 587F 589M 593G 594K 595K 624S 625T 626D 630D 632R 636E 637I 640Q 643D 647K 650N 653R 654F 702L 706Q 709Q 710N 713N 714V 717S 718N 722D 725K 729Y 805R 809N 810R 813E 816R 819K 822G 823K 887E 888R 890W	Homocos

TABLE S3 Summary of structural and evolutionary information of *ileS* SNPs in isoleucyl –tRNA synthetase (IleRS)

		3 D complex information of *ileS* SNPs in IleRS			Evolutionary information[a]
**Mutant**	**ns-SNP(s) in *ileS***	Predicted position<br><br>Exposed/Buried	Predicted secondary Structure	Contacting molecule(s)<br><br>(Direct contact / contact site(s) close by)	**(% of AA among homologous proteins)**
VSV3	A196V	exposed	G(3/10-helix)	Nucleotide	A:69% H:12% S:6% F:5% N:2% T:2% Y:2% D:1% G:1%
VSV2	Y723H	buried	H(alpha-helix)	Isoleucyl-tRNA contact sites close by	I:37% R:17% L:16% V:14% F:7% A:3% M:3% Y:2% N:1% G:1%

[a]Underlined is the wild type occurring residue of parental FDA209P strain's IleRS protein.
